# Interleukin-6^2^/lymphocyte as a proposed predictive index for COVID-19 patients treated with monoclonal antibodies

**DOI:** 10.1007/s10238-023-01081-6

**Published:** 2023-04-25

**Authors:** Salvatore Rotundo, Massimo Borelli, Vincenzo Scaglione, Rosaria Lionello, Flavia Biamonte, Vincenzo Olivadese, Angela Quirino, Helen Linda Morrone, Giovanni Matera, Francesco Saverio Costanzo, Alessandro Russo, Enrico Maria Trecarichi, Carlo Torti, IDTM UMG COVID-19 Group, Francesca Serapide, Bruno Tassone, Paolo Fusco, Chiara Davoli, Valentina La Gamba, Helen Linda Morrone, Lavinia Berardelli, Maria Teresa Tassone, Riccardo Serraino, Chiara Costa, Daniela Patrizia Foti, Federico Longhini, Andrea Bruni, Eugenio Garofalo, Eugenio Biamonte, Domenico Laganà, Maria Petullà, Bernardo Bertucci, Giorgio Settimo Barreca, Aida Giancotti, Luigia Gallo, Angelo Lamberti, Maria Carla Liberto, Nadia Marascio, Adele Emanuela De Francesco

**Affiliations:** 1 Department of Medical and Surgical Sciences, University “Magna Graecia”, Catanzaro, Italy; 2 UMG School of PhD Programmes, University “Magna Graecia”, Catanzaro, Italy; 3 Department of Clinical and Experimental Medicine, University “Magna Graecia”, Catanzaro, Italy; 4 Department of Health Sciences, University “Magna Graecia”, Catanzaro, Italy; 5 Department of Experimental and Clinical Medicine, University “Magna Graecia” of Catanzaro, Catanzaro, Italy; 6 Anesthesia and Intensive Care Unit, Department of Medical and Surgical Sciences, University “Magna Graecia” of Catanzaro, Catanzaro, Italy; 7 Radiology Division, Department of Experimental and Clinical Medicine, University Hospital “Mater Domini”, Catanzaro, Italy; 8 Unit of Hospital Pharmacy, University Hospital “Mater Domini”, Catanzaro, Italy

**Keywords:** Interleukin, Lymphocytes, COVID-19, SARS-CoV-2, Monoclonal antibodies

## Abstract

In a convenience sample of 93 patients treated with monoclonal antibodies (moAbs) against SARS-CoV-2, the interleukin-6^2^/lymphocyte count ratio (IL-6^2^/LC) was able to predict clinical worsening both in early stages of COVID-19 and in oxygen-requiring patients. Moreover, we analysed 18 most at-risk patients with asymptomatic or mild disease treated with both moAbs and antiviral treatment and found that only 2 had clinical progression, while patients with a similar risk were reported to have an unfavourable outcome in most cases from recent data. In only one of our 18 patients, clinical progression was attributable to COVID-19, and in the other cases, clinical progression was observed despite IL-6^2^/LC being above the risk cut-off. In conclusion, IL-6^2^/LC may be a valuable method to identify patients requiring more aggressive treatments both in earlier and later stages of the disease; however, most at-risk patients can be protected from clinical worsening by combining moAbs and antivirals, even if levels of the IL-6^2^/LC biomarker are lower than the risk cut-off.

## Introduction

The spectrum of coronavirus disease 19 (COVID-19) ranges from asymptomatic disease to severe acute respiratory syndrome leading to death [1]. It has been suggested that low lymphocyte count (LC) [2–8] as well as high serum IL-6 level is associated with a worse outcome in COVID-19 [4–6, 9–13], even though these biomarkers are poorly specific and have limited prognostic power. Interestingly, a marker composed of both measures, namely IL-6/LC, appears to be promising because elevated IL-6/LC predicts mortality in both patients with severe (SEV) COVID-19 [14] according to the National Institutes of Health (NIH) classification [15] and in those with respiratory failure not requiring intensive care [16]. To the best of our knowledge, no studies have investigated the role of the IL-6/LC ratio in patients treated with neutralizing monoclonal antibodies (moAbs), especially in the early stage of infection (asymptomatic [ASY] or mild [MID] disease [15]).

The objective of this study was to explore the prognostic value of a modified ratio (IL-6^2^/LC) in a cohort of COVID-19 patients treated with moAbs, both moderate [MOD] or SEV cases and in the early stages. Additionally, by analysing the clinical cases, the possible role of the above marker in relation to the treatment adopted and patient outcome was assessed, with the aim to suggest further implementation of therapeutic strategies in the context of the main problems faced by clinicians today.

## Patients and methods

All patients treated with moAbs in the third-level University Hospital of Catanzaro (Southern Italy) from 27th April 2021 to 1st April 2022 were included in this retrospective study, provided that the serum IL-6 concentration and LC were available at baseline (i.e. before starting treatment including moAbs in all patients tested for IL-6 24 h prior). IL-6 was measured with a non-competitive chemiluminescent immunoassay (Elecsys by Roche). LC was determined by an automatic blood cell counter (ADVIA 2120 by Siemens).

Patients were stratified into two groups: those who received moAbs in the early stage of infection (ASY or MID disease according to NIH [15]) and those already requiring oxygen therapy due to COVID-19 pneumonia (MOD and SEV diseases [15]). Patients affected by a more severe COVID-19 (such as those undergoing high flow nasal cannula or mechanical ventilation) did not receive moAbs, as indicated by the Italian Medicinal Agency (AIFA) regulation. Clinical worsening was the outcome of the study, as defined by any transition to a worse NIH clinical class [15] during follow-up.

Before coming to our centre for treatment (either as outpatients or inpatients), the patients were evaluated by territorial medical services via molecular testing (i.e. real-time polymerase chain reaction [RT–PCR]) or third-generation rapid antigen nasal swab analysis to determine SARS-CoV-2 infection. SARS-CoV-2 infection was further confirmed by RT–PCR (GeneFinder COVID-19 Plus RealAmp Kit, Elitech Group) performed on nasopharyngeal swabs on the same day of hospital admission or moAbs administration. MoAbs were chosen for each patient while taking into consideration the local epidemiological spread of SARS-CoV-2 variants of concern (VOCs) and the likely activity of moAbs. Outpatients, as well as inpatients after discharge, were followed-up until nasopharyngeal swab analysis turned out to be negative (third-generation rapid antigen or molecular test).

The study was approved by the ethical committee of the Calabria Region (protocol reference: FESR/FSE 2014–2020 DDRC n. 4585, Action 10.5.12, noCOVID19@UMG) and was carried out in accordance with the Declaration of Helsinki and the principles of Good Clinical Practice guidelines [17]. Written informed consent was obtained both for the administration of moAbs and antivirals and for using the anonymized patient data for research purposes.

### Statistical analysis

Continuous variables are summarized as the mean and standard deviation (SD); categorical variables are summarized as absolute frequencies and percentages. Fixing the statistical significance at an alpha = 0.05 level both for univariate and multivariate inferential analyses, differences between categorical variables were assessed by Fisher’s exact test and associations were evaluated by odds ratios (ORs). Comparisons of continuous variables were performed by the Student–Welch *t* test or by the Mann–Whitney *U* test, considering each quantitative trait after testing it for normality using the Shapiro–Wilk test. The binomially distributed primary outcome (i.e. worsening condition) was modelled by logistic regression, and the accuracy of predictors was explored by means of receiver operating characteristic (ROC) curve analysis as implemented in the ROCR library [18] of the open source package R [19].

## Results

### Patient characteristics

During the study period, 140 patients (intended sample) were treated with moAbs. 47 patients were excluded from the study due to unavailability of IL-6 levels and/or LC at baseline. Therefore, 93 patients were included in the convenience sample of the study. Table 1 shows the characteristics for both the overall intended sample and the convenience sample. No statistically significant differences in patients’ characteristics were found among the two samples.

**Table 1:** Characteristics of patients treated with moAbs (intended and convenience samples)

Characteristic	Intended sample (*n* = 140)	Convenience sample (*n* = 93)	*p* value
Female (*N*, %)	77 (55%)	53 (57%)	0.766
Age (mean, SD)	65.5 (16.1)	65.4 (16.4)	0.958
Body Mass Index (mean, SD)	28.7 (6.5)	27.9 (5.8)	0.447
Heart failure (*N*, %)	9 (6.4%)	8 (8.6%)	0.534
Cerebral ischaemia history (*N*, %)	17 (12.1%)	13 (1%)	0.423
Chronic obstructive pulmonary disease (COPD) (*N*, %)	25 (17.9%)	17 (18.3%)	0.934
Autoimmune disease (*N*, %)	12 (8.6%)	9 (9.7%)	0.775
Vasculopathy (*N*, %)	17 (12.1%)	13 (14%)	0.684
Hepatic cirrhosis (*N*, %)	1 (0.7%)	1 (1.1%)	0.775
Diabetes (*N*, %)	35 (25%)	22 (23.7%)	0.79
Chronic kidney disease (*N*, %)	15 (10.7%)	12 (12.9%)	0.615
Cancer (*N*, %)	16 (11.4%)	11 (11.8%)	0.951
AIDS (*N*, %)	1 (0.7%)	0 (0%)	0.42
Peptic ulcer (*N*, %)	1 (0.7%)	1 (1.1%)	0.775
Ischaemic heart disease (*N*, %)	22 (15.7%)	19 (20.4%)	0.356
Dementia (*N*, %)	12 (8.6%)	6 (6.4%)	0.555
Hemiplegia (*N*, %)	3 (2.1%)	2 (2.1%)	1
Hypertension (*N*, %)	85 (60.7%)	56 (60.2%)	0.94
Haematology malignancy (*N*, %)	22 (15.7%)	17 (18.3%)	0.609

*SD* standard deviation

### Predictive value of IL-6^2^/LC at baseline for clinical worsening

Among the 93 patients included in the convenience sample, 74 received moAbs at ASY/MID stages, whereas 19 were treated at MOD/SEV stages. The study outcome was reached in 28 patients overall (14 in the ASY/MID group and 14 in the MOD/SEV disease group: 19% versus 74%, OR: 11.6, Fisher’s exact test *p* < 0.001, 95% confidence interval: 3.3–48.3). The mean values of IL-6^2^/LC were 1.12 (SD: 3.18) in patients who did not subsequently worsen and 7.88 (SD: 13.63) in those who did. A statistically significant difference was found when comparing IL-6^2^/LC ratios between the two groups (Mann–Whitney *U* test, *p* < 0.001).

Table 2 shows the significant (*p* = 0.010) predictive value of the IL-6^2^/LC ratio combined with dichotomized clinical classes according to logistic regression with respect to outcome. Additionally, several possible additive and multiplicative models correcting for gender, age, body mass index (BMI), hypertension and combination therapy were explored, never obtaining any further significant term in predicting outcome.

**Table 2:** Predictive value of the IL-6^2^/LC ratio combined with dichotomized clinical classes according to logistic regression analysis

Measure/group	Estimate	Standard error	*z*	*p* value
Intercept	− 2.13	0.4	− 5.31	< 0.001
IL-6^2^/LC	0.59	0.26	2.32	0.02
Oxygen-requiring group	3.08	0.72	4.26	< 0.001
Interaction term	− 0.58	0.26	− 2.25	0.02

Based on the results shown in Table 2, it was possible to consider a further proposed predictive index (ppi) that discriminates the outcome probability stratifying the patient sample into the two aforementioned risk classes, according to the following relationships:

$$\begin{aligned} & \left[ {{\text{low}}\;{\text{risk}},{\text{ ASY}}\;{\text{MID}}} \right] \quad \quad {\text{ppi}}_{{{\text{low}}}} = \, - 2.13 + 0.59*\left( {{\text{IL}} - 6^{2} /{\text{LC}}} \right) \\ & \left[ {{\text{high}}\;{\text{risk}},{\text{MOD}}\;{\text{SEV}}} \right]\quad \quad {\text{ppi}}_{{{\text{high}}}} = \, 0.95 + 0.01*\left( {{\text{IL}} - 6^{2} /{\text{LC}}} \right) \\ \end{aligned}$$

**Fig. 1:**
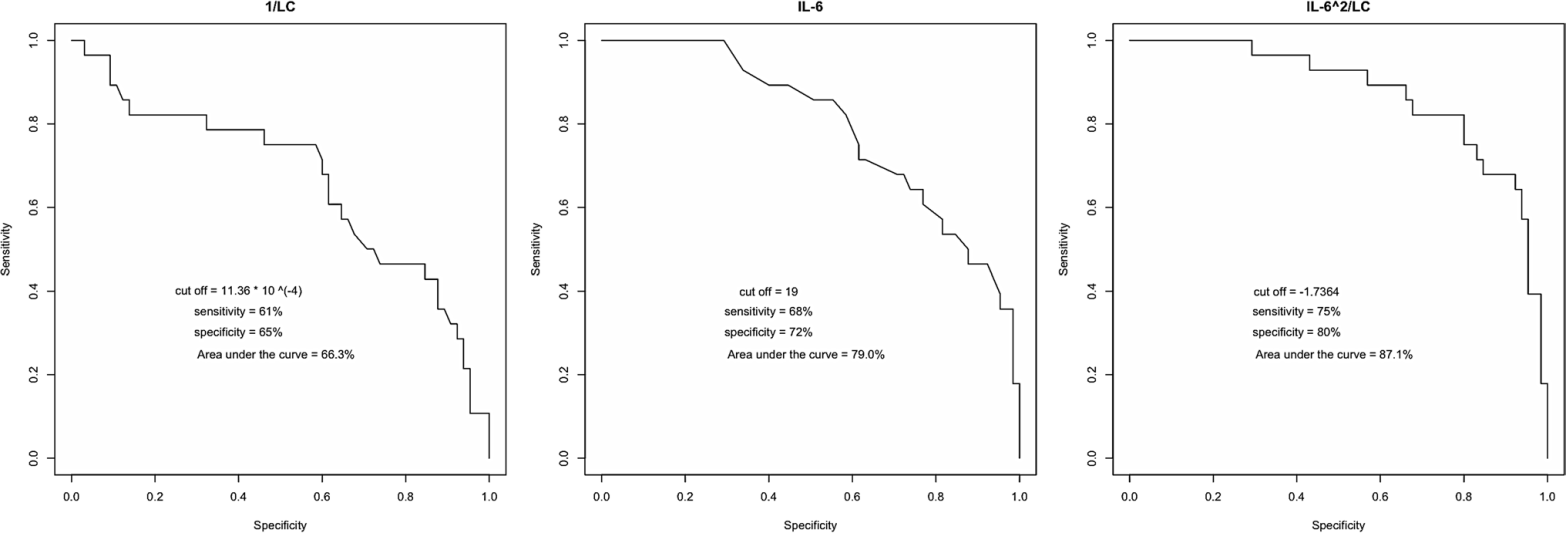
Receiver operating characteristic curves for LC^−1^ (left panel), IL-6 (middle panel) and IL-6^2^/LC (right panel). The reciprocal value of the lymphocyte count (LC^−1^) was analysed instead of LC to preserve graph convexity. IL-6^2^/LC, as distinguished in ppi between ASY/MID and MOD/SEV disease, exhibits superior accuracy and area under the curve value

To further investigate the predictive role of the IL-6^2^/LC ratio, ROC curve analysis was performed to compare the performances of IL-6, IL-1/LC and IL-6^2^/LC for predicting clinical worsening (see Fig. 1).

Table 3 reports the cut-offs, sensitivity, specificity and area under the curve (AUC) for IL-6, IL-1/LC and IL-6^2^/LC ppi.

**Table 3:** Results of analysis of receiver operating characteristic curves for IL-6

Predictor	Cut-off	Sensitivity	Specificity	AUC
IL-6	19	68	72	79
1/LC	11.4 * 10^–4^	61	65	66.3
IL-6^2^/LC ppi	− 1.74	75	80	87.1

1/LC and IL-6^2^/LC ppi

*ppi* proposed predictive index

### Clinical description of patients with ASY/MID disease treated with combination therapy (intended sample) and correlation with IL-6^2^/LC (convenience sample)

In order to explore the prognostic value of IL-6^2^/LC in the subset of patients who received combination therapy, we chose to focus on clinical outcome in patients with ASY/MID disease who fell into this group who displayed a highly specific positive predictive value of the IL-6^2^-LC ratio, probably because the outcome of patients with MOD/SEV disease could be primarily driven by their advanced stage of COVID-19.

In 22 patients of the intended sample (ASY/MID disease) with a very high risk of progression to severe COVID-19 due to incomplete vaccination, immunocompromised status and/or severe and multiple comorbidities, we prescribed combination therapy composed of moAbs and nirmatrelvir/ritonavir 150/100 mg (three pills two times a day for 5 days) or molnupiravir 400 mg (four pills two times a day for five days) or remdesivir short course regimen (200 mg/ev the first day and 100 mg/ev the second and the third day). None of these patients died.

Among these 22 patients treated with combination therapy in the ASY/MID stages, 18 were tested for IL-6 serum levels and LC. IL-6^2^/LC was under the risk cut-off in all except one patient affected by myeloma treated with sotrovimab plus nirmatrelvir/ritonavir, who recovered without reaching the study endpoint. Only 2 of 18 patients reached the study endpoint. The first was a female affected by chronic lymphocytic leukaemia and hypogammaglobulinemia treated with ibrutinib who needed admission due to COVID-19. She required oxygen therapy with low-flow nasal cannula (2 L/min) for three days and then improved until recovery. The second patient was a severe cardiopathic male admitted for implantation of a cardiac resynchronization defibrillator (CRT-D) who developed ventricular tachycardia and respiratory failure during hospitalization not attributable to COVID-19. The remaining 16 patients who did not meet the study endpoint received moAbs for the following indications: 3 patients were not vaccinated against SARS-CoV-2; 7 patients had haematological malignancies, such as lymphoma/lymphatic leukaemia (*n* = 5), myeloma (*n* = 1) or Waldenström macroglobulinemia (*n* = 1); 2 patients had cancer (an elderly female diagnosed as being infected by SARS-CoV-2 a few days after surgery for colon cancer and a middle-aged woman who had ovarian cancer); 2 patients received kidney transplantation and were taking immunosuppressive drugs; 1 patient, a middle-aged male, was affected by systemic lupus erythematosus treated with mycophenolate; 1 patient, a young woman, developed tricuspid endocarditis associated with intravenous drug abuse. Only 4 of the 13 vaccinated patients in this group had detectable IgG against the spike protein of SARS-CoV-2. Last, 5 of the patients with haematological diseases had received anti-CD20 moAbs in the year preceding moAb administration.

For the remaining 4 of 22 patients who received combination therapy and whose IL-6 serum level at baseline was not available, 3 patients had lymphoma and 1 had multiple sclerosis treated with anti-CD20 moAbs. Interestingly, none of these patients reached the study endpoint.

## Discussion

SARS-CoV-2 infection is asymptomatic in at least 40% of cases [20], and vaccination further decreases the risk of clinical progression [21]. However, older patients and those with significant comorbidities or immunodeficiency (including those who receive immunosuppressive drugs [22]) are at greater risk of clinical progression despite vaccination. Several studies have demonstrated that moAbs targeting SARS-CoV-2 epitopes are able to reduce the risk of clinical complications in patients with mild or moderate COVID-19 who did not receive previous vaccination [23–26]; therefore, moAbs were introduced into clinical practice following emergency approval. However, the effectiveness of moAbs for the treatment of immunocompromised patients is unclear and no studies have investigated the possible role of biomarkers to predict which patients may experience clinical progression despite moAb treatment.

Herein, we describe results from a real-life study on a group of patients treated with moAbs who had heterogeneous features with regard to vaccination status and comorbidities (including immunosuppression). As expected, patients presenting with advanced COVID-19 were more likely to reach the study outcome, indicating disease progression. However, 14/74 (19%) patients in the convenience sample had clinical deterioration even though they were treated in the first 5 days from symptom onset during the ASY/MID stages. This highlights the need to identify the most at-risk patients who may need more effective strategies for treatment and follow-up to improve their outcomes.

With this objective in mind, we explored the possible predictive value of the IL-6^2^/LC ratio both in patients with ASY/MID and in those with MOD/SEV disease. The predictive value of this biomarker is supported by biologic plausibility because IL-6 and LC appear to be linked by an inverse proportionality relationship, which supports the biological rationale for a greater predictive value of IL-6^2^/LC compared to that of IL-6 and LC separately. In fact, IL-6 is produced by different lymphocyte subsets [27] and, in turn, stimulates its own production through paracrine mechanisms while decreasing blood LC [28] and facilitating adhesion of lymphocytes to the endothelium and migration into lymphoid tissues [29].

The main finding of the present study was that the IL-6^2^/LC was able to predict clinical worsening both in early stages of COVID-19 and in oxygen-requiring patients. In particular, in patients with ASY/MOD COVID-19, the IL-6^2^/LC ratio appeared to be a highly specific predictive worsening index because for a unit increase in the ratio, the ppi increased by 0.59. This finding is of potential relevance in clinical practice because patients with IL-6^2^/LC ratio greater than the risk cut-off may be candidate to receive more effective treatments to prevent clinical evolution even though they are in the ASY/MID stages of the disease.

Although recommendations are not yet supported by data [15], it has been hypothesized that most at-risk patients should receive combination therapy including both moAbs and antivirals [30–32]. For this reason, we treated our patients using a flow chart of drug prescription choices according to risk of progression of COVID-19, including combination therapy in those with high risk of severe disease [31]. In our former study [31], we included all patients treated with antivirals and/or moAbs, while in the present analysis, we selected only patients treated with moAbs, with or without antivirals, for whom IL6 and LC were available to derive the novel biomarker.

When focusing on patients with ASY/MID disease treated with combination therapy, we found that most patients belonging to this convenience sample displayed IL-6^2^/LC below the risk cut-off except for one, who recovered without reaching the study end-point. It is not possible to conclude whether outcome was improved as an effect of combination therapy or whether the low IL-6^2^/LC value in these patients was indeed a predictor of a better clinical outcome. However, the following considerations support the first hypothesis in this subgroup of patients: (i) despite treatment with moAbs or antivirals alone, immunosuppressed patients may show more prolonged infections, leading to unfavourable clinical outcomes [33]; (ii) other studies suggested an advantage in administering combination therapy to COVID-19 patients either in preventing death [34] or in shortening time to discharge home, remaining alive for at least 14 days [35]; (iii) in our former study [31], none of the most at-risk outpatients treated with combination therapy were subsequently admitted to hospital for COVID-19, while four immunocompromised patients who received only moAbs or an antiviral drug developed a more severe disease resulting in hospital admissions. Therefore, we suggest that, in our study, combination treatment contributed to achieving a favourable outcome despite high risk of disease progression, even in one patient whose IL-6^2^/LC ratio was above the risk cut-off. It may also be hypothesized that in these patients the predictive value of the IL-6^2^/LC ratio is diluted by a greater risk of progression, implying that they should be more aggressively treated notwithstanding the IL-6^2^/LC values. Anyway, an elevated IL-6^2^/LC ratio remains predictive of unfavourable outcome even in an exploratory multivariable model, corrected for use of combination treatment. This may indicate that the study marker is suited in the overall population of patients to guide therapeutic choices, although its impact for tailoring of treatment might be more important in patients with a lower risk of disease progression.

There are several limitations to this study. First, it is a single-centre retrospective study using a convenience sample, which limits the generalizability of the results. Second, the sample size was small and although an attempt at performing a multivariate analysis was made, this analysis must be considered to be merely explorative; in other terms, the absence of an evidence in this analysis does not mean evidence of an absent impact or correlation of the outcome with the given factors among those tested (such as gender, age, BMI, hypertension and combination therapy) [36]. Third, the chosen outcome (i.e. transition to more severe classes according to NIH classification of the disease [15]) was not as strong as death, implying that our conclusions should be de-emphasized until further studies will confirm them using more relevant outcomes. Fourth, the study population was heterogeneous and we could not further stratify patients by comorbidities or any other factors of interest due to the limited sample size. Therefore, since our target population is widely heterogeneous, patients with haematological, cardiovascular, and autoimmune disorders, and the COVID-19 population category should be more appropriately selected. Among the many factors which may have an impact on the results, one most relevant is immunosuppression, either primary or secondary, since the immunocompromised population during COVID-19 may or may not mount an inflammatory response [38, 39]; in this setting, the LC could be altered due to both underlying conditions (such as treatment with immunosuppressive drugs [40]) and COVID-19 [2–8]. Finally, the study relied on a single biomarker (IL-6^2^/LC ratio) to predict clinical worsening, which may not be sufficient to capture all relevant factors influencing disease progression. To address this limitation, future studies could include multiple biomarkers or other clinical measures to predict clinical worsening. Since this study is explorative in nature, we decided to avoid any comparisons with other possible biomarkers [37]. Moreover, since IL-6^2^/LC was available only at baseline for each patient, we could not assess whether possible variations of this marker during the follow-up were correlated with the clinical course as already performed in a previous study using different biomarkers [37]. Therefore, while the present study findings are valuable, there are several limitations that could be addressed in the future studies to enhance the generalizability and applicability of the findings.

## Conclusions

In conclusion, the IL-6^2^/LC ratio appears to predict clinical worsening in SARS-CoV-2-positive patients treated with neutralizing moAbs both in the early stages of the infection and, although in a less sensitive manner, in COVID-19 patients requiring oxygen. However, more powerful studies should be conducted using multivariable models to adjust for possible confounding variables. Moreover, prospective, randomized studies are needed to confirm whether interventions with new treatment strategies (for example, combining antivirals with moAbs) will further improve patient outcomes when used at earlier stages of the disease under the guide of new biomarkers. For instance, if the predictive value of the IL-6^2^/LC ratio in the overall patient population and in patients stratified by significant features is confirmed, patients in these studies should be randomized after stratification by levels of IL-6^2^/LC. In the meantime, we suggest that IL-6^2^/LC is useful to strictly monitor patients to provide more proactive assessment, treatment and support.

## Data Availability

The datasets used and/or analysed during the current study available from the corresponding author on reasonable request.

## References

[1] Maier HE, Kuan G, Saborio S (2021). Clinical spectrum of SARS-CoV-2 infection and protection from symptomatic re-infection. Clin Infect Dis.

[2] Huang I, Pranata R (2020). Lymphopenia in severe coronavirus disease-2019 (COVID-19): systematic review and meta-analysis. J Intensive Care.

[3] Huang W, Berube J, McNamara M (2020). Lymphocyte subset counts in COVID-19 patients: a meta-analysis. Cytom A.

[4] Izcovich A, Ragusa MA, Tortosa F (2020). Prognostic factors for severity and mortality in patients infected with COVID-19: a systematic review. PLoS ONE.

[5] Terpos E, Ntanasis-Stathopoulos I, Elalamy I (2020). Hematological findings and complications of COVID-19. Am J Hematol.

[6] Velavan TP, Meyer CG (2020). Mild versus severe COVID-19: laboratory markers. Int J Infect Dis.

[7] Illg Z, Muller G, Mueller M, Nippert J, Allen B (2021). Analysis of absolute lymphocyte count in patients with COVID-19. Am J Emerg Med.

[8] Biamonte F, Botta C, Mazzitelli M (2021). Combined lymphocyte/monocyte count, D-dimer and iron status predict COVID-19 course and outcome in a long-term care facility. J Transl Med.

[9] Galvan-Roman JM, Rodriguez-Garcia SC, Roy-Vallejo E (2021). IL-6 serum levels predict severity and response to tocilizumab in COVID-19: an observational study. J Allergy Clin Immunol.

[10] Herold T, Jurinovic V, Arnreich C (2020). Elevated levels of IL-6 and CRP predict the need for mechanical ventilation in COVID-19. J Allergy Clin Immunol.

[11] Van Singer M, Brahier T, Ngai M (2021). COVID-19 risk stratification algorithms based on sTREM-1 and IL-6 in emergency department. J Allergy Clin Immunol.

[12] Gorham J, Moreau A, Corazza F (2020). Interleukine-6 in critically ill COVID-19 patients: a retrospective analysis. PLoS ONE.

[13] Trecarichi EM, Mazzitelli M, Serapide F (2020). Clinical characteristics and predictors of mortality associated with COVID-19 in elderly patients from a long-term care facility. Sci Rep.

[14] Yang B, Chang X, Huang J (2021). The role of IL-6/lymphocyte ratio in the peripheral blood of severe patients with COVID-19. Int Immunopharmacol.

[15] In Coronavirus Disease 2019 (COVID-19) Treatment Guidelines: Bethesda (MD), 2021. 34003615

[16] Masotti L, Grifoni E, Pelagalli G (2022). Prognostic role of Interleukin-6/lymphocytes ratio in SARS-CoV2 related pneumonia. Int Immunopharmacol.

[17] World Medical A (2013). World medical association declaration of Helsinki: ethical principles for medical research involving human subjects. JAMA.

[18] Sing T, Sander O, Beerenwinkel N, Lengauer T (2005). ROCR: visualizing classifier performance in R. Bioinformatics.

[19] Al Bander Z, Nitert MD, Mousa A, Naderpoor N (2020). The Gut microbiota and inflammation: an overview. Int J Environ Res Public Health.

[20] Oran DP, Topol EJ (2020). Prevalence of asymptomatic SARS-CoV-2 infection: a narrative review. Ann Intern Med.

[21] Accorsi EK, Britton A, Fleming-Dutra KE (2022). Association between 3 doses of mRNA COVID-19 vaccine and symptomatic infection caused by the SARS-CoV-2 omicron and delta variants. JAMA.

[22] Calderon-Parra J, Munez-Rubio E, Fernandez-Cruz A (2022). Incidence, clinical presentation, relapses and outcome of severe acute respiratory syndrome Coronavirus 2 (SARS-CoV-2) infection in patients treated with anti-CD20 monoclonal antibodies. Clin Infect Dis.

[23] Dougan M, Nirula A, Azizad M (2021). Bamlanivimab plus etesevimab in mild or moderate Covid-19. N Engl J Med.

[24] Weinreich DM, Sivapalasingam S, Norton T (2021). REGN-COV2, a neutralizing antibody cocktail, in outpatients with Covid-19. N Engl J Med.

[25] Gupta A, Gonzalez-Rojas Y, Juarez E (2021). Early treatment for Covid-19 with SARS-CoV-2 neutralizing antibody sotrovimab. N Engl J Med.

[26] Montgomery H, Hobbs FDR, Padilla F (2022). Efficacy and safety of intramuscular administration of tixagevimab-cilgavimab for early outpatient treatment of COVID-19 (TACKLE): a phase 3, randomised, double-blind, placebo-controlled trial. Lancet Respir Med.

[27] Chen Q, Wang WC, Bruce R (2004). Central role of IL-6 receptor signal-transducing chain gp130 in activation of L-selectin adhesion by fever-range thermal stress. Immunity.

[28] Majidpoor J, Mortezaee K (2022). Interleukin-6 in SARS-CoV-2 induced disease: interactions and therapeutic applications. Biomed Pharmacother.

[29] Evans SS, Repasky EA, Fisher DT (2015). Fever and the thermal regulation of immunity: the immune system feels the heat. Nat Rev Immunol.

[30] Cohen MS (2022). Early treatment to prevent progression of SARS-CoV-2 infection. Lancet Respir Med.

[31] Scaglione V, Rotundo S, Marascio N (2022). Lessons learned and implications of early therapies for coronavirus disease in a territorial service centre in the Calabria region: a retrospective study. BMC Infect Dis.

[32] D’Abramo A, Vita S, Nicastri E (2022). The unmet need for COVID-19 treatment in immunocompromised patients. BMC Infect Dis.

[33] Scherer EM, Babiker A, Adelman MW (2022). SARS-CoV-2 evolution and immune escape in immunocompromised patients. N Engl J Med.

[34] ACTIV-3–Therapeutics for Inpatients with COVID-19 (TICO) Study Group. Tixagevimab-cilgavimab for treatment of patients hospitalised with COVID-19: a randomised, double-blind, phase 3 trial. Lancet Respir Med 2022;10:972–984. 10.1016/S2213-2600(22)00215-6 10.1016/S2213-2600(22)00215-6 PMC9270059 35817072

[35] Group A-TBS, Lundgren JD, Grund B, et al. Responses to a Neutralizing Monoclonal Antibody for Hospitalized Patients With COVID-19 According to Baseline Antibody and Antigen Levels: A Randomized Controlled Trial. Ann Intern Med 2022;175:234–243. 10.7326/M21-3507 10.7326/M21-3507 PMC9334931 34928698

[36] Riley RD, Cole TJ, Deeks J (2022). On the 12th day of Christmas, a statistician sent to me. BMJ.

[37] Solimando AG, Susca N, Borrelli P (2020). Short-term variations in Neutrophil-to-Lymphocyte and Urea-to-Creatinine ratios anticipate intensive care unit admission of COVID-19 patients in the emergency department. Front Med.

[38] Remy KE, Mazer M, Striker DA (2020). Severe immunosuppression and not a cytokine storm characterizes COVID-19 infections. JCI Insight.

[39] Liu BM, Hill HR (2020). Role of host immune and inflammatory responses in COVID-19 cases with underlying primary immunodeficiency: a review. J Interferon Cytokine Res.

[40] Furlan A, Forner G, Cipriani L (2021). COVID-19 in B cell-depleted patients after rituximab: a diagnostic and therapeutic challenge. Front Immunol.

